# Health status and air pollution related socioeconomic concerns in urban China

**DOI:** 10.1186/s12939-018-0719-y

**Published:** 2018-02-05

**Authors:** Kaishan Jiao, Mengjia Xu, Meng Liu

**Affiliations:** 1 Department of Sociology, Minzu University of China, 27 Zhongguancun South Avenue, Beijing, 100081 China; 2 Department of Economics, Claremont Graduate University, 170 E. 10th Street, Claremont, CA 91711 USA; 3 Department of Social Work, China Women’s University, 1 Yuhui Dong Lu, Chaoyang District, Beijing, 100101 China

**Keywords:** Health inequality, Air pollution, Mediation, Urban China

## Abstract

**Background:**

China is experiencing environmental issues and related health effects due to its industrialization and urbanization. The health effects associated with air pollution are not just a matter of epidemiology and environmental science research, but also an important social science issue. Literature about the relationship of socioeconomic factors with the environment and health factors is inadequate. The relationship between air pollution exposure and health effects in China was investigated with consideration of the socioeconomic factors.

**Methods:**

Based on nationwide survey data of China in 2014, we applied the multilevel mixed-effects model to evaluate how socioeconomic status (represented by education and income) contributed to the relationship between self-rated air pollution and self-rated health status at community level and individual level.

**Results:**

The findings indicated that there was a non-linear relationship between the community socioeconomic status and community air pollution in urban China, with the highest level of air pollution presented in the communities with moderate socioeconomic status. In addition, health effects associated air pollution in different socioeconomic status groups were not equal. Self-rated air pollution had the greatest impact on self-rated health of the lower socioeconomic groups. With the increase of socioeconomic status, the effect of self-rated air pollution on self-rated health decreased.

**Conclusions:**

This study verified the different levels of exposure to air pollution and inequality in health effects among different socioeconomic groups in China. It is imperative for the government to urgently formulate public policies to enhance the ability of the lower socioeconomic groups to circumvent air pollution and reduce the health damage caused by air pollution.

## Background

Environmental pollution has attracted great attention in China due to its rapid development of industrialization and urbanization in recent years. The report of city smog has widely occurred in the mass media in China [1]. The social implication of air pollution has indicated an increasing number of severe problems of air pollution in China^1^ and the health effects associated with air pollution. Air pollution may cause acute or chronic health problems including mild irritation to the upper respiratory tract, chronic respiratory system disease, heart disease, lung cancer, children’s acute respiratory infection and adults’ chronic bronchitis. In addition, air pollution exacerbates asthma, heart disease or lung disease, and increases the risk of death [2, 3]. Meanwhile, health effects caused by air pollution are significantly different for people with different socioeconomic statuses. Even though Beck argued that poverty was hierarchical and chemical smog was democratic [4], some researches have pointed out that individuals and groups with different socioeconomic statuses were exposed to air pollution at different levels and suffered from different health effects. These differences refer to environmental inequality and health inequality.

Some studies have examined the health inequalities associated with air pollution at regional level. For example, a study of six districts in Sao Paulo, Brazil, demonstrated that PM10 had less effect on respiratory mortality among older adults in areas with a higher proportion of college education populations and high-income families, and it had greater effect in areas where the proportion of the poor population was high [5]. Another study based on the city of Hamilton in Canada showed that air pollution exposure had a relatively large effect on acute mortality in the zones with a higher proportion of low educational attainment and high manufacturing employment [6]. In addition, a study of Hong Kong, China, found that air pollution had a greater effect on the mortality of people living in public rental than those living in private homes, and a greater effect on blue-collar workers than those who had never worked and white-collar workers [7]. Other studies focusing on developed areas, such as Rome and Norway, revealed similar conclusion that neighborhood socioeconomic deprivation could exacerbate mortality caused by air pollution [8, 9].

On the other hand, some studies have examined the health inequalities associated with air pollution at individual level. For example, a study of 20 cities in the United States showed that individual education significantly modified the relationship between PM10 and mortality; specifically, the higher education level of the individual the lower the effect of PM10 on mortality [10]. In addition, several studies about China found similar results that people with lower socioeconomic status normally experienced a higher health risk from air pollution, while people with middle or high socioeconomic status barely had a health risk since they had more ways to avoid air pollution [11]. Moreover, as the severity of air pollution increased, health disparities among people with different socioeconomic status would intensify [12]. On the contrary, a good natural environment could reduce health inequality [13].

There are two possible reasons that explain the stronger effect of air pollution among people in low socioeconomic class. First, the level of exposure to air pollution is higher among those living in low socioeconomic communities [14]. Pearce and Kingham claimed that in some countries or regions the socially deprived, the low income and the ethnic minority were exposed to a higher level of air pollution [15]. Furthermore, Schoolman and Ma found that townships with higher proportion of rural migrants were exposed to higher level of air pollution in Jiangsu province, China [16]. Second, compared to people in high socioeconomic status, populations with low socioeconomic status are more susceptible to air pollution. The susceptibility of people with low socioeconomic status is caused by health-related social, behavioral and psychological factors including poor health status (such as diabetes and obesity), addictions (such as smoking), other pollutant exposures (such as passive smoking), psychological stress, low intake of nutrition, and even genetic make-up [17].

Forastiere pointed out that the second reason (i.e. different susceptibility of individuals) was more convincible than the first one (i.e. differential exposure to air pollution) for explaining the stronger effect of air pollution among low socioeconomic groups [8]. This is because several researchers have found no significant difference in levels of exposure in air pollution among people with different socioeconomic status, and some even have reached the opposite conclusion that high socioeconomic groups experience high levels of air pollution. For example, Goodman et al. examined the traffic-related air pollution in London and found that even though the level of average air pollution concentrations was higher in low socioeconomic positions, reversed direction of association was expected to occur in central London area [18]. Similar results were found in studies by Crouse and Cesaroni et al. [19, 20]. People with a college education or living in high socioeconomic communities were more likely to be exposed to traffic-related air pollution. Havard et al. asserted that the relationship between air pollution exposure and deprivation was nonlinear and the medium level deprivation areas were the most exposed to air pollution [21]. Thus, the association between socioeconomic status and air pollution exposure was still under uncertainty and required further investigation [22]. However, it should be noted that several studies did not find a modification effect of socioeconomic status on air pollution [23], or they found just a partial impact that education modified the effect of air pollution but household income did not [6]. Some researchers even found opposite results, i.e. the effect of air pollution on mortality was higher among people with high socioeconomic status [24]. Therefore, Laurent et al. argued that the modification effect of socioeconomic status depended on the regional level at which socioeconomic characteristics were measured [17]. If socioeconomic characteristics were measured at city level, no modification effect was found; if at community level, the result was mixed; if individually measured socioeconomic characteristics were used, the result indicated disadvantaged subjects were affected more by pollution.

To sum up, although existing studies have found that people with different socioeconomic status experienced varying levels of exposure to air pollution and health effects, unanimous conclusion has not been reached. In addition, most of the previous studies have focused on developed countries or regions. Therefore, studies need to be further extended to developing countries like China, which is now at a stage of accelerated industrialization and urbanization. Study of air pollution and health inequality in China can provide new empirical evidence for the debate. Therefore, this study tried to extend the literature with the aim to explore whether there was a significant difference in air pollution exposure and health effects among different socioeconomic status individuals or communities in China. To be more specific, there are two sub-questions: (1) Is the level of exposure to air pollution different among communities with different socioeconomic status in China, and what happens to the level of air pollution exposure as the community’s socioeconomic status continues to improve? (2) Is the effect of air pollution on health associated with socioeconomic status? With the increase of socioeconomic status, either at community level or at individual level, does the effect of air pollution on health decrease?

## Methods

### Data

The data of this study was collected from “China Labor Dynamics Survey”(CLDS) [25], which is China’s first national longitudinal social survey on its labor force. Since 2012, this survey has been conducted every two years. The CLDS data includes individuals, families and communities, covering 29 cities in China (except for Hong Kong, Macao, Tibet and Hainan). Based on the purpose of this study, subjects living in urban communities were chosen because air pollution occurred mainly in the city, and air pollution in rural areas was relatively insignificant and less of a concern. Several previous studies were also based on the city sample [26–29]. In addition, the current study selected participants over 45 years old because the impact of air pollution on health was mainly concentrated on children and older people, who were also the main target of many studies. The final sample size was 3838 individuals from 171 urban communities.

### Variables and measurement

#### Self-rated health

Although some studies pointed out that self-rated health had some limitations over objective health markers [30, 31], some researchers explained in details the unique characteristics of self-rated health and its role in predicting mortality [32–34]. Unlike most of the health indicators, self-rated health is based on a subjective cognitive process [35], which captures the overall subjective experience of mental and physical well-being, and is closer to the WHO’s definition of health [36]. Additionally, several studies have found self-rated health had a high reliability. For example, Lundberg and Manderbacka assessed the reliability of self-rated health and found that the self-rated health was reliable in all subgroups studied, and the reliability of self-rated health was even excellent in the group of older men [37]. Consistent with some health surveys [38, 39], self-rated health in this study is based on the answers to the question of ‘how do you evaluate your own health conditions’, and five different levels of answers: very good (coded as 1), good (coded as 2), moderate (coded as 3), bad (coded as 4) and very bad (coded as 5).

#### Community health status

To examine the relationship between community air pollution and the overall health of the community, we collected the self-rated health scores of all the individuals in a given community to calculate the measure of community health status. This had been done by calculating the percentage of respondents who reported ‘very good’ and ‘good’ in self-rated health in each community. The higher the percentage, the better the health condition in the community.

#### Self-rated air pollution

In epidemiology and environmental science, the measurement for air quality is normally the concentration of pollutant (such as submicron particles, particulate matter (PM), ozone (O_3_), nitrogen dioxide (NO_2_), carbon monoxide (CO), and sulfur dioxide (SO_2_)), which is an objective index. It should be pointed out that the air quality index of a city is a summary of the data of multiple air monitoring stations in this city. Given the relatively small number of air monitoring stations and short-term air pollution data in China’s cities [40], it is questionable whether the officially released Air Quality Index (AQI) can reflect the true level of air pollution. Moreover, the size of China’s cities is relatively large, and the levels of air pollution in different communities within a city can be significantly different [41]. Therefore, a single AQI does not necessarily reflect the true level of air pollution exposure of community inhabitant. According to Forastiere and Galassi, the best source of learning about a fact, at least in theory, was from the most informed subject who was experiencing the event, especially when his/her report reflected several aspects [42]. In fact, in some studies about community characteristics and health, subjective perception of air quality was seen as an important feature of the community [43–45]. Furthermore, some studies have found that perceived pollution and health risks played an important role in understanding the health effects of air pollution [46]. In addition, when studying the effects of air pollution, psychological factors should be taken into account [47]. Compared with other kinds of pollution that was not easily perceived by community residents, air quality was more easily perceived [48–50]. Therefore, to some extent, self-rated air pollution can reflect the level of effect of air pollution on individuals who are being exposed to it. In this study, self-rated air pollution is based on the answers to the question of ‘how do you rate the level of air pollution in the place where you live’. The answers from subjects were categorized into two levels: not serious air pollution (coded as 1) and serious air pollution (coded as 2).

#### Community air pollution level

The percentage of number of subjects who reported ‘serious air pollution’ in that community was calculated as the air pollution index of that community. The larger the percentage, the more severe the perceived air pollution was in the community.

#### Individual socioeconomic status (SES)

Education, occupation and income are the common measurements for socioeconomic status, but some studies argued that such measurement was mainly for developed countries and debated whether it fitted developing countries [51, 52]. This study focused on the population over 45 years old, most of whom were not working, so the variable of occupation on this population was null. Considering the relationship between education and income and the completeness of the data, this study did not include occupation as measurement for socioeconomic status. For measuring socioeconomic status, this study introduced two indicators – years spent in school (*E*_*i*_) and per capita annual household income (*I*_*i*_). As shown in Table 1, according to the number of years specified in each stage of education in China, we converted education level to the years spent in school. The per capita annual household income was calculated by dividing the annual income of the household by the number of members in that household. We first standardized the years spent in school: *Z*_*i*1_ = (*E*_*i*_ − *mean*)/*Standard deviation*, and standardized the per capita annual household income: *Z*_*i*2_ = (*I*_*i*_ − *mean*)/*Standard deviation*. Then, we calculated the mean of two standardized variables as the individual’s socioeconomic status: *SES*_*i*_ = (*Z*_*i*1_ + *Z*_*i*2_)/2.

**Table 1:** Years of Education Transformed from Level of Education

Level of Education	Years Spent in School	
	Graduated	Not Graduated
Never at School	0	0
Primary School	6	3
Middle School	9	7.5
High School	12	10.5
Vocational High School	12	10.5
Technical School	12	10.5
Technical Secondary School	12	10.5
College	15	13.5
University (Bachelor)	16	14
University (Master)	19	17.5
University (PhD)	22	20.5

#### Community socioeconomic status (CSES)

The CSES was calculated as the mean of the individual’s socioeconomic status in each community: *CSES*_*j*_ =  ∑ *SES*_*ij*_/*n*_*j*_.

#### Covariates

The covariates in this study included age, gender, marriage, body mass index, smoking, drinking and physical exercise, which may significantly influence health. Age was a continuous variable, and gender was a dummy variable (with female coded 1, male coded 0). Marriage was categorized as single (coded 1) or married (coded 0). Body mass index was categorized into four classifications: light weight (coded 1), normal weight (coded 2), overweight (coded 3) and obesity (coded 4). Smoking was categorized into three classifications: never smoke (coded 1), quit smoking (coded 2), and always smoke (coded 3). Drinking (includes white wine, red wine and beer) was also categorized into three classifications: never drink (coded 1), quit drinking (coded 2), and always drink (coded 3). Physical exercise was a dummy variable: not often physical exercise (coded 1) and often physical exercise (coded 0). Table 2 shows the measurement of variables and their description.

**Table 2:** Description of variables (N/% or mean)

Variables		N	%
*Individual characteristics*			
Gender			
	Male	1793	46.72
	Female	2045	53.28
Marriage			
	Single	3554	92.6
	Married	284	7.4
Body mass index			
	Light weight	283	7.37
	Normal weight	2420	63.05
	Overweight	803	20.92
	Obesity	332	8.65
Smoking			
	Never smoke	2758	71.86
	Quit smoking	185	4.82
	Always smoke	895	23.32
Drinking			
	Never drink	3095	80.64
	Quit drinking	90	2.34
	Always drink	653	17.01
Physical exercise			
	Not often	1486	38.72
	Often	2352	61.28
Self-rated health			
	Very good	574	14.96
	Good	1514	39.45
	Moderate	1278	33.3
	Bad	414	10.79
	Very bad	58	1.51
Self-rated air pollution			
	Not serious	2694	70.19
	Serious	1144	29.81
		N	Mean
Age		3838	54.45
Socioeconomic Status		3838	0.36
*Community characteristics*			
Community health status		172	63.95
Community Socioeconomic Status		172	0.56
Community Air Pollution Level		172	31.35

### Model

Considering the multilevel structure (individual < family < community < city) of the data and the advantages of multilevel model [53, 54], this study applied the multilevel mixed-effects model. For continuous responses, this study used linear mixed effects model; for ordinal responses, this study used generalized linear mixed-effects model.

First, we set up a model to examine the relationship between the community air pollution exposure and the community socioeconomic status. The model was specified as:

1 $$ {y}_{jk}={\beta}_0+{\beta}_1{x}_{1 jk}+{\beta}_2{x}_{1 jk}^2+{u}_k+{e}_{jk} $$

where the subscript *k* denotes city (level-2), *j* denotes community (level-1), *y*_*jk*_ is community air pollution exposure, *x*_1*jk*_ is community socioeconomic status, *u*_*k*_ is level-2 random effects, and *e*_*jk*_ is level-1 random effects.

Second, we set up a model to examine the relationship between the community health status and the community air pollution exposure. The model was specified as:

2 $$ \kern2em {y}_{jk}={\beta}_0+{\beta}_1{x}_{1 jk}+{\beta}_2{x}_{2 jk}+{\beta}_3{x}_{1 jk}{x}_{2 jk}+{\beta}_4{x}_{3 jk}+{u}_k+{e}_{jk} $$

where the subscript *k* denotes city (level-2), *j* denotes community (level-1), *y*_*jk*_ is community health status, *x*_1*jk*_ is community socioeconomic status, *x*_2*jk*_ is community air pollution exposure, *x*_1*jk*_*x*_2*jk*_ is the interaction term of community socioeconomic status and community air pollution exposure, *x*_3*jk*_ is the proportion of elder population in the community, *u*_*k*_ is level-2 random effects, and *e*_*jk*_ is level-1 random effects.

Finally, we set up a generalized linear mixed-effects model to examine the relationship between socioeconomic status, self-rated air pollution and self-rated health at individual level. The model was specified as:

3 $$ \mathrm{logit}\left(\frac{y_{ijk}>m}{y_{ijk}\le m}\right)={\beta}_0+{\beta}_1{x}_{1 ijk}+{\beta}_2{x}_{2 ijk}+{\beta}_3{x}_{1 ijk}{x}_{2 ijk}+\kern0.5em {\sum}_{q=4}^Q{\beta}_q{x}_{qijk}+{u}_k+{u}_{jk} $$

where the subscript *k* denotes community (level-3), *j* denotes household (level-2), *i* denotes individual, *y*_*ijk*_ is self-rated health, *x*_1*ijk*_ is self-rated air pollution exposure, *x*_2*ijk*_ is individual socioeconomic status, *x*_1*ijk*_*x*_2*ijk*_ is the interaction term of self-rated air pollution exposure and individual socioeconomic status, *x*_*qijk*_ is covariate, *u*_*jk*_ is level-2 random effects and *u*_*k*_ is level-3 random effects.

The models were run using the *meglm* command in *Stata Statistical Software* for maximum likelihood estimation. For comparing the nested models, the -2LL, i.e. the deviance statistic, was used for significance test. The smaller the deviation, the better the model. The difference of deviations from two models was under the chi-square distribution with degree of freedom equaling the difference of numbers of parameters in each model.

## Results

First, we examined the relationship between community socioeconomic status, community air pollution and community health. As shown in Table 3, the community socioeconomic status was positively related to community air pollution. As the community socioeconomic status increased, the level of community air pollution rose simultaneously. However, the coefficient of squared community socioeconomic status was significantly negative (*P* < 0.01), which indicated a curved relationship between community socioeconomic status and community air pollution. In other words, it was possible that the relationship between community socioeconomic status and community air pollution was mediated by community socioeconomic status.

**Table 3:** Community socioeconomic status, community air pollution and community health

	Community air pollution	Community health status
Intercept	21.540*** (2.508)	70.374*** (3.309)
Community Socioeconomic Status	16.305*** (3.951)	2.302 (2.642)
Community Socioeconomic Status × Community Socioeconomic Status	−5.254*** (1.896)	
Community Air Pollution		−0.243** (0.095)
Community Socioeconomic Status × Community Air Pollution		0.141* (0.076)
Proportion of Elder Population		−0.364*** (0.128)
Variance of Level 2 (City)	151.437*** (42.540)	30.371 (18.927)
Variance of Level 1 (Community)	233.359*** (30.198)	193.240*** (24.776)

***p < 0.01, **p < 0.05, *p < 0.1; Standard errors are in brackets

**Fig. 1:**
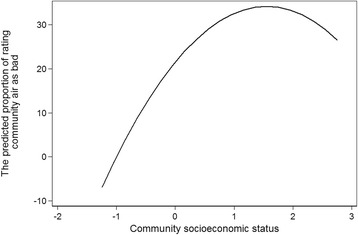
Community socioeconomic status and community air pollution

Figure 1 presents the predicted values of community air pollution (percentage of serious air pollution assessment in community samples) at different levels of community socioeconomic status based on the models in Table 3. It can be seen that as community socioeconomic status improved, air pollution in community continued to increase. However, when community socioeconomic status increased to a certain extent, air pollution in community declined as community socioeconomic status increased.

**Fig. 2:**
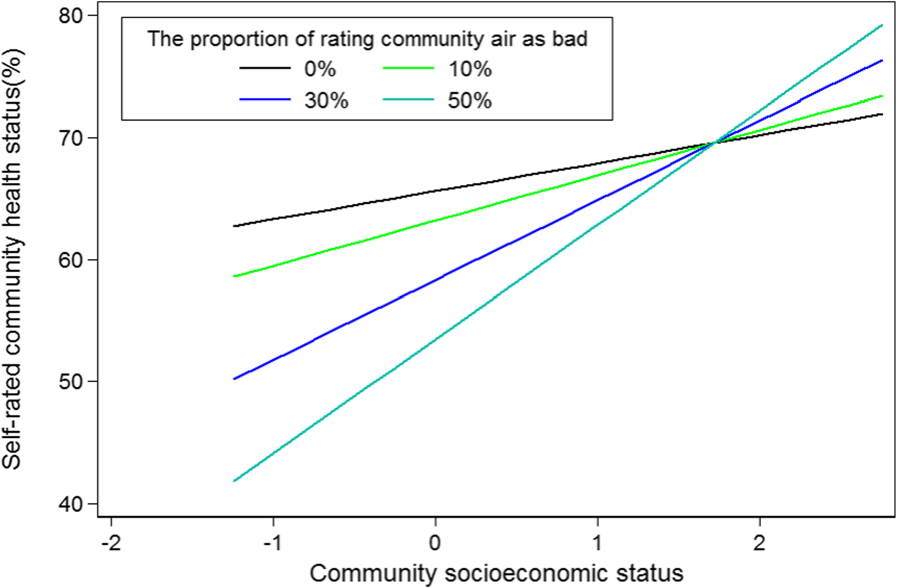
Self-rated community air pollution and community health status: mediated by socioeconomic status

Additionally, Table 3 demonstrates a negative relationship between community air pollution and community health, i.e. when the level of air pollution in community increases, the health at community level will decline. Table 3 also shows the coefficient of the interaction term is 0.141 (*P* < 0.1), which means that health effects associated with community air pollution were mediated by community socioeconomic status. As community socioeconomic status increased, the effect of community air pollution on community health decreased. As Fig. 2 illustrates, among the communities with low socioeconomic status, the more serious the air pollution, the worse the health conditions in the community. However, as the socioeconomic status increased, the difference of health conditions among communities with different levels of air pollution shrunk. That is to say, air pollution had relatively small impact on health in communities with higher socioeconomic status. It was important to point out that in communities with very high socioeconomic status, the health status of communities with higher air pollution was better than that of communities with relatively low air pollution.

In addition, we also examined the relationship between self-rated air pollution and self-rated health, and the mediating role of individual socioeconomic status. Table 4 shows the results of the mixed effects model. Model 1 in Table 4 contained only control variables and model 2 added self-rated air pollution. According to the likelihood ratio test results of the two models, model 2 (χ^2^(1)=24.11, *P* = 0.000) was better than model 1, which indicated self-rated air pollution significantly affected self-rated health. The more severe the self-rated air pollution of the community where the individual is living, the worse self-rated health will be. In model 3, individual socioeconomic status variables were added. Likelihood ratio test results showed model 3 (χ^2^(1)=89.47, *P* = 0.000) was better than model 2, which indicated individual socioeconomic status was significantly related to self-rated health. Individuals with higher socioeconomic status were significantly less likely to have bad self-rated health status than those with lower socioeconomic status. In model 4 of Table 4, we have added an interaction term of individual socioeconomic status and self-rated air pollution. Likelihood ratio test results showed model 4 (χ^2^(1)=4.76, *P* = 0.029) was better than model 3, which indicated the health effects associated with self-rated air pollution were mediated by individual socioeconomic status. The coefficient of the interaction term was −0.247, which was statistically significant (*P* < 0.05). Thus, with the improvement of individual socioeconomic status, the impact of self-rated air pollution on self-rated health would be significantly reduced.

**Table 4:** Relationship between Individual socioeconomic status, self-rated air pollution and self-rated health

Variable (Reference)	Model 1	Model 2	Model 3	Model 4
*Fixed Parameter*				
Female (Male)	0.262*** (0.095)	0.257*** (0.095)	0.154 (0.095)	0.158* (0.095)
Age	0.057*** (0.007)	0.057*** (0.007)	0.047*** (0.007)	0.047*** (0.007)
Single (Married)	0.305** (0.151)	0.337** (0.151)	0.302** (0.150)	0.305** (0.150)
Not often physical Exercise (often)	0.187** (0.085)	0.198** (0.085)	0.082 (0.086)	0.083 (0.086)
Quitted Smoking (Never Smoke)	0.319* (0.192)	0.314 (0.192)	0.311 (0.192)	0.315 (0.192)
Smoking (Never Smoke)	0.003 (0.117)	−0.006 (0.117)	−0.044 (0.117)	−0.040 (0.117)
Quitted Drinking (Never Drink)	1.117*** (0.259)	1.093*** (0.259)	1.052*** (0.259)	1.039*** (0.259)
Drinking (Never Drink)	−0.385*** (0.117)	−0.381*** (0.117)	−0.370*** (0.117)	−0.369*** (0.117)
Normal Weight (light weight)	−0.739*** (0.155)	−0.739*** (0.155)	−0.694*** (0.154)	−0.691*** (0.154)
Overweight (light weight)	−0.713*** (0.169)	−0.710*** (0.169)	−0.672*** (0.168)	−0.668*** (0.168)
Obesity (light weight)	−0.507*** (0.194)	−0.506*** (0.194)	−0.474** (0.194)	−0.467** (0.193)
Serious Self-Rated Air Pollution (not serious)		0.483*** (0.099)	0.511*** (0.099)	0.614*** (0.110)
Individual Socioeconomic Status			−0.558*** (0.061)	−0.482*** (0.069)
Serious Self-Rated Air Pollution × Individual Socioeconomic Status				−0.247** (0.113)
*Random Parameter*				
Variance of Level 3 (Community)	0.850*** (0.134)	0.836*** (0.133)	0.766*** (0.124)	0.772*** (0.125)
Variance of Level 2 (Family)	1.657*** (0.232)	1.651*** (0.231)	1.584*** (0.224)	1.570*** (0.223)
Sample Size	3838	3838	3838	3838
Log Likelihood	−4753	−4741	−4696	−4694
Degree of Freedom	11	12	13	14

***p < 0.01, **p < 0.05, *p < 0.1; Standard errors are in brackets

**Fig. 3:**
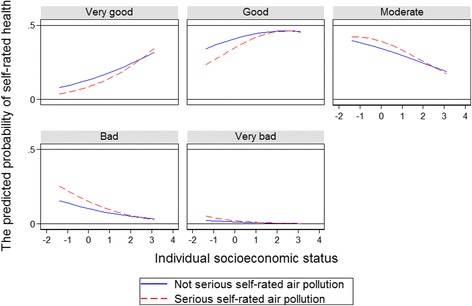
Self-rated air pollution and self-rated health: mediated by socioeconomic status

As Fig. 3 illustrates, among individuals with low socioeconomic status, those who rated air pollution as ‘serious’ were less likely to rate health as “very good” or “good” than those who rated air pollution as ‘not serious’. However, as the level of individual socioeconomic status increased, the difference of health effects caused by self-rated air pollution decreased. From Fig. 3, among individuals with high socioeconomic status, the predicted probability of self-rated health as ‘very good’ and ‘good’ among those who rated air pollution as ‘serious’ was very close to that among those who rated air pollution as ‘not serious’. Figure 3 also implies that individuals rating air pollution as ‘serious’ were more likely to report ‘moderate’, ‘bad’ and ‘very bad’ for health status than those rating air pollution as ‘not serious’, especially among those with lower socioeconomic status. Similarly, among individuals with higher socioeconomic status, the predicted probability of self-rated health as ‘moderate’, ‘bad’ and ‘very bad’ among those who rated air pollution as ‘serious’ was very close to that among those who rated air pollution as ‘not serious’. In short, the level of individual socioeconomic status was negatively correlated to the health effects associated with self-rated air pollution.

## Discussion

This study found a non-linear relationship between community air pollution and community socioeconomic status in urban China. On the one hand, as community socioeconomic status improves, air pollution in the community is also on the rise, which, to some extent, indicates air pollution is a ‘byproduct’ of economic development of the society. This finding is consistent with some previous research. For example, in cities such as London and Rome, communities with high socioeconomic status had a higher level of exposure to air pollution [18, 20]. However, when the community socioeconomic status was raised to a certain level, the relationship between it and community air pollution began to reverse. In other words, as community socioeconomic status further increased, community air pollution would decrease. It implied that air pollution exposure was the highest among the communities with moderate socioeconomic status [21], while communities with lowest or highest socioeconomic status experienced relatively less air pollution.

In general, we can classify the relationship between community socioeconomic status and air pollution into five categories: low community socioeconomic status - low community air pollution (Class 1), moderate community socioeconomic status - moderate community air pollution (Class 2), high community socioeconomic status - high community air pollution (Class 3), high community socioeconomic status - moderate air pollution (Class 4) and high community socioeconomic status - low community air pollution (Class 5). At present, most urban areas in China are at the stage of rapid socioeconomic development and increasing air pollution (Class 1). Whether these urban areas transform from Class 2 to Class 3 or from Class 2 to Class 4 depends on changes in the economic development mode and investment in air pollution control. Additionally, some cities are at the stage of high socioeconomic status and high air pollution (Class 3), such as Beijing and Tianjin. With the change of economic development mode and the increase of government’s investment in air pollution control, air pollution in these cities like Beijing will continue to decline in the future (from Class 3 to Class 4). Finally, some cities in China have higher socioeconomic status but lower air pollution (Class 4 or Class 5), such as Shenzhen and Xiamen.

The factors to be considered in explaining the relationship between community socioeconomic status and community air pollution include economic structure, industrialization patterns, urbanization patterns, the natural environment and individual choices. While higher socioeconomic groups tend to prefer communities with better air quality, this is not the case in most of China’s cities. Now when urban residents choose their living location, they consider work opportunity, educational resources, medical resources and transportation more than air quality. Because of the homogeneity of density of air pollution in a city, such as PM2.5 [55, 56], individuals have to move to another city if they want to live in a community with better air quality. Considering the communities with serious air pollution are usually featured by highly developed economics, abundant educational and medial resources, and convenient transportation in China, people with high socioeconomic status do not demonstrate strong selection preference for high-level air quality. Therefore, high socioeconomic groups experience higher level of air pollution exposure in China. However, if air pollution becomes worse or its health risks are perceived to become higher, higher socioeconomic people are likely to make air quality an important consideration for relocation, such as moving from air polluted areas to areas with good air quality. If this becomes the case, the result will be that higher socioeconomic people will live in communities with good air quality, while the lower socioeconomic people will live mostly in poor air quality communities.

In addition, this study also found that there were significant inequalities in health effects associated with air pollution. With the improvement of socioeconomic status, the health effects associated with air pollution had been declining. As a result, the differences in self-rated health among people living in areas with severe air pollution and those living in areas with less polluted air had been gradually reduced. To be more specific, the health effects associated with air pollution on lower socioeconomic people was significantly greater than that of higher socioeconomic people. The result may be explained by vulnerability and avoidance of people with different socioeconomic status [14]. First, compared with higher socioeconomic people, the health status of lower socioeconomic groups is relatively poor [57], making them more sensitive to severe air pollution and more vulnerable to health damage. Second, people with lower socioeconomic status have significantly less access to good quality medical services than those with higher socioeconomic status [58], such that they cannot receive medical services in a timely manner when air pollution aggravates and affects their health. Third, people with low socioeconomic status usually have relatively low capability of prevention. Even under the same level of air pollution, people with low socioeconomic status are exposed into air pollution more due to work environment (for example, outdoor) and indoor environment (for example, the low capability of protection against air pollution). Lastly, because of the low educational background, people with low socioeconomic status usually lack the knowledge of health and environmental pollution as well as the health effects caused by air pollution. Therefore, they are more likely to have lower level of awareness of self-protection.

Because people with low socioeconomic status are less likely to be well protected from air pollution, they may experience more health effects than those with high socioeconomic status. Given the lack of capacity of lower socioeconomic groups to avoid the risk of air pollution, the public service of air pollution prevention provided by the government is very important. At present, when the air is heavily polluted, the main task of the government is to reduce pollutant emissions. Policies related to air pollution risk management are seriously lagging behind, resulting in lower socioeconomic people (such as children from poor families, the elderly, patients, etc.) failing to receive the related public services. Therefore, it is imperative for the regions with more serious air pollution to urgently formulate public policy to mitigate the negative impact of air pollution exposure. The main goals of public policy makers should be to increase the government’s financial investment to provide masks, air purification equipment and other protective means and information consulting services for lower socioeconomic people. In addition, the government should continue to improve existing health care policies, enhance the accessibility of high-quality medical services to the lower socioeconomic people, and minimize the health damage caused by air pollution.

This study has some limitations as follows. First, due to the limited number of air quality monitoring stations in China, only the city-level data was available rather than community-level data. Therefore, air pollution was measured in a subjective way in this study. Although some studies suggested that subjectively perceived community characteristics (such as perception of community air quality) played an important role in health research [59], and some studies also found that perceived air pollution was significantly correlated with official monitoring data [60], more effort should be on the relative role of objective air pollution and subjectively perceived air pollution in influencing health conditions. Additionally, whether the differences in the impact of air pollution on the health status of different socioeconomic people are caused by the differences in perceptions of air pollution also requires further study. Second, since this study used cross-sectional data, there is no in-depth study on causal mechanisms between socioeconomic status, air pollution and health status. Future studies may consider using panel data to examine how changes in socioeconomic status affect exposure to air pollution and how changes in air pollution exposure affect health. Third, although the self-rated health used in this study contained the evaluation of one’s own overall health status as well as WHO’s definition of health, the impact of air pollution on some objective health indicators needs further investigation. The association between air pollution and different health indicators, and whether this relationship is modified by socioeconomic status also requires further investigation. Finally, since the sample of this study only included urban residents over the age of 45, the conclusions of this study cannot be generalized to the large population. We need to extend the study of the health effects associated with air pollution in the general population and the mediating role of socioeconomic status. Moreover, we still need to elaborate the difference in different subgroups such as age group differences.

## Conclusion

This study used nationwide multilevel data to investigate how socioeconomic factors matter in the relationship between air pollution and health status in China. It verified the different levels of exposure to air pollution and inequality in health effects among different socioeconomic groups in China. The findings indicated a nonlinear relationship between community socioeconomic status and community air pollution, and the highest level of the relationship was found in communities with moderate socioeconomic status. It was also found that air pollution had the greatest impact on the health of the lower socioeconomic groups. With the increase of socioeconomic status, the effect of air pollution on health was decreased. Therefore, it is imperative for the government to urgently formulate public policies to enhance the ability of the lower socioeconomic groups to circumvent air pollution and reduce the health damage caused by air pollution. On account of the dynamic evolution of socioeconomic development and level of air pollution, the modification effect on the relationship between environment and health from the socioeconomic status is changing. Therefore, it is important to further investigate and analyze the inequality of exposure to environmental pollution and health effects associated with air pollution in China.

## Footnotes

1 According to the Report on the State of the Environment in 2015 released by the Ministry of Environmental Protection of China, among the 338 prefecture-level cities in China only 73 (21.6%) cities had qualified air quality and 265 (78.4%) cities failed the air quality test.
